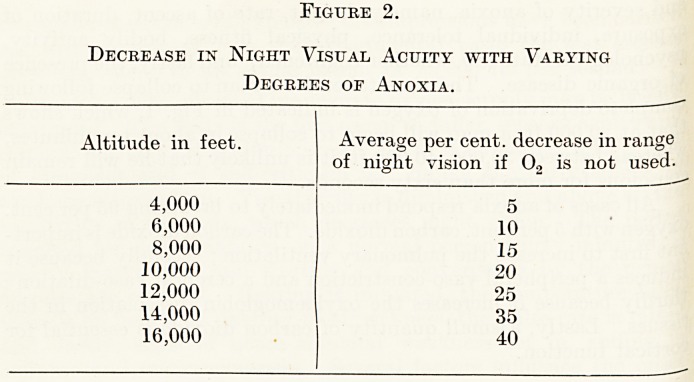# The Thirty-Fifth Long Fox Memorial Lecture: Some Aspects of Aviation Medicine

**Published:** 1947

**Authors:** K. G. Bergin


### The Bristol Medico-Chirurgical Journal

*" Scire est nescire, nisi id me*
*Scire alius sciret."*

SUMMER, 1947.

### THE THIRTY-FIFTH LONG FOX MEMORIAL LECTURE

BY

K. G. BERGIN, M.A., M.D., A.F.R.Æ.S.

*A.D.M.S., B.O.A.C.*

DELIVERED IN THE UNIVERSITY OF BRISTOL ON NOVEMBER 19th, 1946

ON

### SOME ASPECTS OF AVIATION MEDICINE

EVEN in the first World War it was realized that flying performance was not only dependent on methods of propulsion or structural stresses of air-frames, but on the limitations of the human system. Before the onset of the second World War, the Royal Air Force, appreciating this, approved the formation of the Institute of Aviation Medicine at Farnborough, where the Royal Aeronautical Establishment for aerodynamic and other aviation research is situated. The idea is to have physiological and medical research carried on by doctors in immediate contact with all flying problems as they arise. Many of the doctors are themselves experienced pilots. The Institute of Aviation Medicine proved of immense value during the war. With the advent of peace civil aviation needs similar help.

### RESPIRATION

*Atmospheric Changes.* Problems of the respiratory system are more numerous than those of other systems by reason of the fact that flying takes place largely in atmospheric conditions differing from those encountered at ground level. At sea level atmospheric pressure is 760 mm. Hg. or 14.6 lbs. to the square inch. At 20,000 ft. the atmospheric pressure is 350 mm. Hg. or 7 lbs. to the square inch, and at 40,000 ft. 141 mm. Hg. or 3 lbs. to the square inch. The atmospheric pressure decreases with height, and the percentage composition of alveolar air also changes, because the partial pressure of water vapour and carbon dioxide remains constant through very great variations of pressure, thereby affecting the ratio of the other gases in the lungs. For example, when the partial pressure of inspired oxygen is two-thirds of that at sea level, the partial pressure of alveolar oxygen is one-half ; when the partial pressure of inspired oxygen is one-seventh, that of alveolar oxygen is one-twenty-third, and at a height of 50,313 ft., where the pressure of inspired oxygen is 86 mm. Hg., the pressure of alveolar oxygen is nil.

Various compensating mechanisms come into play during ascent to a decreased atmospheric pressure. In aviation the ascents to height are rapid and do not permit of the compensatory adjustments which occur in slow ascents such as in mountaineering: in these cases there is an increase in the number of red blood cells, increased ventilation rate and changes in the tissue oxidase system. The desirable objective in flying is to maintain the alveolar oxygen at a pressure of 70–100 mm. Hg.

*Oxygen Systems.* This result is obtained by supplying oxygen to the aviator by a number of different systems:

The "Free Flow System" consists of a reservoir of oxygen under pressure, a delivery pipe and an oxygen mask allowing a steady flow of oxygen to the pilot, adequate for his use at all altitudes. This system is wasteful of oxgyen and should only be used in the single-seater, short endurance aircraft. Its great virtue is its simplicity.

In the "Reservoir (or Rebreathing) System" the oxygen passes first into a low-pressure reservoir fitted with a rebreather bag, into which the user expires. Unused oxygen in the exhaled air is, therefore, available for future use. By means of a system of valves the pilot only utilizes such oxygen as he actually requires for each particular height, with great economy in oxygen. Such systems are used on all heavy types of aircraft and in the majority of civil airlines to-day.

"Pressure Breathing." These systems fail to deal with the problems of oxygen supplies at heights greater than 33,500 ft., and the presence of German reconnaisance aircraft at heights exceeding 40,000 ft., early in 1943, called for a lighter device than a pressurized cabin. Over 40,000 ft. pure oxygen is insufficient to maintain adequate alveolar oxygen pressure. Oxygen had to be introduced at a positive pressure by means of a reservoir designed as a waistcoat giving oxygen at the pressure required for these heights. In practice this was found to be 8 in. of water or 15 mm. of mercury. Such an apparatus was designed and used with great success in 1942–43, increasing the upper altitude tolerance of pilots from 33,000 to 45,000 ft. The use of the apparatus needed practice, but no particular difficulties were encountered. It had to be understood, for example, that in this system expiration became an *active,* and inspiration a *passive* process, contrary to normal breathing; and while a good fit was necessary to avoid leakage, care had to be taken not to interfere with the venous return. It was found advantageous if users of pressure breathing apparatus breathed pure oxygen for thirty minutes before flying.

In "Pressure Cabins" air is introduced by means of a mechanically driven impeller so as to maintain a constant pressure in the cabin of the aircraft irrespective of the pressure outside. If air is introduced at an additional pressure of approximately 5½ lbs. to the square inch, sea-level conditions can be maintained in the cabin when the aircraft is at 13,000 ft. and at 25,000 ft. the air pressure inside the cabin equals that outside at 8,000 ft. Attention must be paid to the maintenance of the correct temperature and humidity of the air, regular changing of the air in the cabin, and the elimination of unpleasant and obnoxious odours. Pressure systems have largely overcome these obstacles, and the aircraft which now fly the Atlantic and other long distances are so fitted that a passenger flies without discomfort at 30,000 ft. or more. The fatigue experienced in long flights is much less in pressurized than non-pressurized cabins.

*Explosive Decompression.* When pressurized cabins were first introduced, concern was expressed as to what might happen to the occupants of such a cabin if, from enemy action or other cause, the pressure suddenly fell; it was feared that serious injury to the human organism might result. Explosive decompression tests were conducted with special experimental decompression chambers. It was found that, apart from transient symptoms, no serious harm occurred provided oxygen was readily available for the subsequent anoxia. Medical officers who were subjected to these tests noted the following symptoms: a feeling of severe pressure on the ears, which quickly disappeared if the Eustachian tubes were patent; abdominal distension, relieved by the expulsion of flatus; slight dyspnoea and cough with mucous expectoration, and transient retrosternal pain. All these symptoms suggest slight trauma to the pulmonary alveoli, but no after effects have been discovered and many thousands of explosive decompressions have been carried out without trouble. In the experimental cases the test ascents were equivalent to an actual ascent of 35,000 ft. per second.

*Anoxia.* The effects of inadequate oxygen supply may be briefly mentioned. The brain is the first organ to suffer, sensory acuity and cerebral activity being diminished. There is euphoria, with a lack of judgment and of self-criticism not unlike the early stages of alcoholic intoxication. Visual acuity is diminished and above all the sensitivity of the rods, which decreases night vision. The period required for dark adaption, in a normal person thirty minutes, is greatly lengthened, and fixation and convergence by the ocular muscles are reduced. Hearing is impaired, the temperature falls, there is an increased liability to air sickness, the pulse rate is raised and, lastly, signs of neuro-muscular weakness set in, ending in collapse and unconsciousness. Several factors influence the onset

FIGURE 1.

Effects produced by complete deprivation of oxygen with varying height—time relationships.

and severity of anoxia, namely, height, rate of ascent, duration of exposure, individual tolerance, physical fitness, bodily activity, psychological state (*e.g.* whether frightened), and lastly, the presence of organic disease. The time taken for a man to collapse following complete deprivation of oxygen is indicated in Fig. 1, which shows that at 20,000 ft. a man will begin to collapse in about ten minutes, whereas between 30,000–40,000 ft. it is unlikely that he will remain conscious for more than sixty seconds.

All cases of anoxia respond immediately to breathing 95 per cent. oxygen with 5 per cent. carbon dioxide. The carbon dioxide is important first to increase the pulmonary ventilation; secondly because it induces a peripheral vaso-constriction and a cerebral vaso-dilation; thirdly because it increases the oxyhæmoglobin dissociation in the tissues. Lastly, a small quantity of carbon dioxide is essential for cortical function.

### VISION

Night vision assumed great importance in the war. At first our own night fighters required it for defence against the German attacks on this country; later it was essential, when the circumstances were reversed, for the allied night-bombing offensive. Vision at night is achieved by means of the rods, of which the greatest concentration is found immediately adjacent to the macula about 10 to 15 degrees from the centre.

Visual purple essential for vision by night is produced in the presence of vitamin A and in the absence of light. Full activation of visual purple is attained after thirty minutes dark adaptation and results in over a thousandfold increase in sensitivity of the rods. Dark adaption can be lost by the briefest exposure to light; so a considerable number of experiments was carried out during the war to determine what light was least likely to interfere with visual purple, the idea being to use this colour for essential lighting of instruments, etc. Ultimately an orange-red, standardized by the National Physical Laboratory, was adopted. A pilot who has been using orange-red lighting in the cockpit for some hours can be assured of almost instantaneous full night vision on looking outside the aircraft. It is interesting to note that the blue lights, as used at the entrance to air-raid shelters at the beginning of the war, were clearly visible from the air at night. Lack of oxygen greatly diminishes night visual acuity and, as the table shows, there is 40 per cent. decrease at a height of only 16,000 ft. (Fig. 2). The range of night vision, of course, varies according to the intensity of light available, which itself depends upon weather conditions, stars, moon, etc. On a dark starlight night an aeroplane can be seen at 700–900 ft., whereas on a night with a full moon reflected over cloud the range is increased to between 5,000–7,000 ft.

FIGURE 2.

DECREASE IN NIGHT VISUAL ACUITY WITH VARYING DEGREES OF ANOXIA.

| Altitude in feet. | Average per cent. decrease in range of night vision if $O_2$ is not used. |
|---|---|
| 4,000 | 5 |
| 6,000 | 10 |
| 8,000 | 15 |
| 10,000 | 20 |
| 12,000 | 25 |
| 14,000 | 35 |
| 16,000 | 40 |

Since the greatest concentration of rods is 10–15 degrees from the centre of the retina, optimum night visual acuity is obtained by off-centre viewing and this constituted an important part of the training of aircrews. Many a night fighter has been spotted by utilization of this principle. It is also important to teach methodical survey of the field of search. A simple geometrical plan was devised by which a person could survey the whole area within thirty seconds in a methodical manner without leaving any part unsearched.

Measurement of night visual acuity has been attempted, but to date no satisfactory method has been found.

Caffeine has a definite stimulating effect upon night vision, but the results are difficult to assess at present. Benzedrine sulphate had a generally stimulating effect, but night vision was not noticeably improved.

*Rod Scotometry.* Varying sized scotomata have been observed in different cases of night visual deficiency. In the normal subject, a central scotoma approximately 4 degrees across will be found in the macular area where no rods are present. Larger scotomata will be observed in cases where night vision is deficient by reason of conditions such as vitamin A deficiency, alcohol, smoking, fatigue or organic disease.

*Ariboflavinosis.* An interesting condition was observed in 1942 in pilots and aircrews whose duties kept them for long periods subjected to a high intensity of light, as when flying over the sea, snow or desert. The symptoms noted were excessive lachrymation, photophobia, eyestrain and a gritty sensation under the lids. Associated symptoms included headaches and decreased visual acuity. Careful ophthalmic examination with a slit lamp revealed one feature common to all cases, namely, the invasion of the cornea by leashes of new and abnormal blood vessels. Now the cornea receives its oxygen supply not directly from the blood stream, but by means of riboflavine. It was conjectured that if there is deficiency of riboflavine, a compensatory vascularization of the cornea occurs. It was, therefore, decided among other things to increase the riboflavine intake of all sufferers, and at the same time to reduce, as far as possible,the direct impact of bright light on their eyes, since riboflavine is destroyed in the presence of light. The normal riboflavine requirements of an adult are 2–3 mgm. daily, and it was found that a daily intake of 5 mgm. cleared the condition up in about six weeks. Subsequent eye examinations revealed a gradual retraction and diminution in the new blood vessels and a complete restoration of normal visual function. Later investigation has thrown doubt on the reliability of corneal vascularization as evidence of ariboflavinosis. Care must be taken to rule out other deficiencies which, if severe, cause corneal vascularization. However, the condition was important and the results of treatment with riboflavine were satisfactory.

### EAR, NOSE AND THROAT.

*Otitic Barotrauma*, alternatively known as traumatic aerotitis, was a grave cause of disability among aircrew during the war. The mechanism of the Eustachian tube in equalizing the pressure in the middle and outer ear is well known. When the external atmospheric pressure is lowered there is a passive opening of the orifice, but the reverse does not hold true. That is to say, when it is required to open the tube during a rise of external atmospheric pressure, active voluntary movement is necessary. Failure to equalize may be due to rapid descent as, for example, when an aircraft is falling down out of control or is diving steeply to avoid attack : a man may be asleep, or unconscious in a descending aircraft and so does not perform the necessary voluntary movements. Or there may be oedema of the tube due to infection, chronic obstruction or stenosis from infection or trauma. When the Eustachian tube fails to open and to equalize pressure in middle and outer ears, the tympanic membrane takes the strain with varying results. The symptoms of the condition are pain and deafness with occasional tinnitus and vertigo. Four clear cut stages may be observed : (1) a slight indrawing of the membrane ; (2) definite indrawing of the membrane with engorgement of the blood vessels ; (3) ruptured vessels, usually in the postero-inferior quadrant ; and (4) rupture of the membrane. In all cases there is inability on the part of the patient to inflate the ear through the Eustachian tube. The condition is largely preventable and active measures may greatly diminish its incidence. First, no one should be permitted to fly at height who has a cold. Admittedly many do so without harm, but by far the greatest number of cases were caused by ignoring this rule. Secondly, persons should be instructed how to inflate the Eustachian tube voluntarily (Valsalva's manœuvre). When once the condition is established, several methods of cure may be tried: (1) Either re-ascent in an aircraft, or, more simply decompression in a decompression chamber, immediately relieve the condition; (2) Inflation by Valsalva's method (not always possible if oedema or other conditions are present), by Politzer's, or by Eustachian catheterization ; (3) Myringotomy with a fine-pointed hypodermic needle. The relief of pain and deafness is dramatic and sudden: the membrane returns to its normal position, and usually, only a spot of mucus remains to mark the point of puncture. If the above methods fail, or the condition has been present too long, nothing but masterly inactivity will restore a damaged drum. This may take from forty-eight hours in a mild case to seventeen days in a severe one.

*Aero-Sinusitis*. The main symptom of this condition is acute pain over the affected sinus. The affected side is often tender to touch and opaque to X-rays. The cause is uncertain : it is probably a blocking of the sinus opening causing imprisonment of air within a closed cavity. A sudden fall in external pressure results in ischaemia which, when followed by engorgement, causes a sub-mucosal hæmorrhage. This, in turn, produces stripping of the mucous membrane and the formation of a polyp-like swelling. The treatment consists of radiant heat, adequate drainage and vaso-constrictors.

*Aviation Deafness*. Audiometric investigations on a number of aircrew show deterioration in auditory acuity, particularly in the range of 4,096 double vibrations per second. Aircraft noises consist of a number of primary tones together with over-tones and under-tones, but the greatest intensity is at 110–115 d.v. /s. Conversational level ranges between 300–3,000 d.v. /s. Otosclerosis has been demonstrated in some patients and nerve trauma in others. The effects of repeated exposure tend to be cumulative, producing permanent alterations in the higher frequency loss. Deafness tends to get worse with age and with increased flying time.

The Lecturer next dealt with the effects of speed and acceleration. Speed seems not to affect persons in an enclosed cabin. Acceleration may be linear or rotational : only the latter is of importance. The rate of rotation determines the centrifugal force, and this may become so great that the circulating blood is driven into the legs and feet, with resultant anoxia of the brain and loss of consciousness—" black-out ". Suitable posture in the aircraft, and pressure suits which raise the external pressure on the legs, diminish these effects. Decompression sickness and air sickness present no special features. Attention must be given in selecting aircrew to psychological as well as physical conditions. Fatigue in flying is important now that long flights are frequent. Elimination of noise and vibration, adequate oxygen supply and correct posture help to avoid undue fatigue. He continued :

### TRANSPORT OF INFANTS AND INVALIDS.

The majority of long-distance flying is done between 10,000 and 30,000 ft. so as to obtain the advantage of settled meteorological conditions and higher operating efficiency for the aircraft : at these heights, changes in atmospheric pressure are important. At heights of less than 10,000 ft., many of the problems discussed do not arise.

*Infants.* Experience shows that, on the whole, infants tolerate flying better than adults. Thus, they are less susceptible to air sickness and to minor decrease of anoxia. A word of warning is necessary, however : if symptoms of anoxia *do* manifest themselves, deterioration is more rapid than in adults unless oxygen is given immediately. The simplest method to ensure that a child equalizes the pressure in the middle and outer ear during descent from height is to insist that it is fed at this time.

*Pregnancy.* There appears to be no greater risk of precipitating labour in air travel than in other forms of transport, except in the roughest possible weather, a risk which the passenger and public carrier must decide for themselves. Pregnant women are usually carried up to and including the eighth month of pregnancy in this country ; in America up to within seventy-two hours of the expected date of delivery ; most internal American airlines can land at a nearby airfield if the need should arise.

*Medical Contra-indications.* Certain cases should not travel by air. The majority consists of those affected by respiratory and cardiac diseases. Pneumothorax is contra-indicated for two reasons : the reduced pulmonary ventilating area would result in an earlier onset of anoxia than would otherwise be the case, and the lowered atmospheric pressure at height results in mediastinal displacement. In pulmonary tuberculosis, the increased liability to hæmoptysis from lowered atmospheric pressure is a contra-indication to flying. Those suffering from pleural effusions, heart disease, anaemia and leukaemia and thyrotoxicosis are barred because of the decreased adaptability to the effects of lowered alveolar oxygen tension. Many other individual cases require special consideration. Thus, peptic ulcer with a previous history of hæmatmesis should be regarded with reserve and each case judged on its merits.

*Specific Fevers.* The advent of air travel has greatly complicated the problem of international quarantine measures and carriage of such diseases. Thus, a person may be in a yellow-fever endemic area one day and in a clear area the following day. This has raised acute international problems for the International Convention of Aerial Navigation, to which all countries subscribe and which regulates the preventative measures to be taken by all travellers by air.

### BIBLIOGRAPHY.

Armstrong, H. G., *Principles and Practice of Aviation Medicine*, Williams & Wilkins, Baltimore, 1939.

Armstrong, H. G., and Heim, J. W., " Effects of Repeated Daily Exposures to Anoxaemia," *Journ. Aviation Med.*, 1938, ix, 92–96.

Barcroft, J., " The Respiratory Function of the Blood. Part 1," *Lessons from High Altitudes*, Cambridge (Eng.) Univ. Press, 1925.

Bauer, L. H., *Aviation Medicine*, Williams & Wilkins, Baltimore, 1926.

Barach, A. L., McFarland, R. A., and Seitz, C. P., " The Effects of Oxygen Deprivation on Complex Mental Functions," *Journ. Aviation Med.*, 1937, viii, 197–207.

Bert, P., *La Pression Barométrique*, Masson et Cie, Paris, 1878.

Benzinger, T., " Das Verhalten der Alveolarluft bei abnehmendem Sauerstoffgehalt der Einatmungsluft and und bei Zasatz von Kohlensaure," *Luftfahrtmed*, i, 326–337, 1937.

Bills, A. G., " Blocking in Mental Fatigue and Anoxaemia Compared," *L. Exp. Pyschol.*, 1937, xx. 437–452.

Cusick, P. L., Benson, O. O., and Boothby, W. M., " Effects of Anoxia and High Concentration of Oxygen on the Retinal Vessels," *Proc. Staff Meet., Mayo Clin.*, 1940, xv, 500–502.

Dickson, E. D. D., McGibbon, J. E. G.. and Campbell, A. C. P., " Acute Otitic Barotrauma," *Laryng. & Otol.*, 1944, lix, 267.

Dill, D. B., and Zamcheck, N., " Respiratory Adjustments to Oxygen-lack in the Presence of Carbon-Dioxide," *Amer. Journ. Physiol.*, 1940, cxxix, 47–52.

Diringshoefen, Heinz von, *Medical Guide for Flying Personnel*, trans. by V. E. Henderson, Univ. of Toronto Press, Toronto, 1940.

Diringshoefen, H. von, and Hartmann, H., " Altitudes Effects of Carbon Monoxide," *Lufttahrt Med.*, 1935, xii. 121, 23.

" Diet, Oxygen Want and High Flying," *Brit. Med. Journ.*, 1940, i, 57.

Feldman, J. B., " Practice of Dark Adaptation," *Arch. Ophth.*, 1938, xix, 882–901.

Gillespie, R. D., *Salmon Lectures*, New York, 1941.

Grow, M. C., " A Study of Fatigue," *Mil. Surgeon*, 1936, lxxviii, 103–119.

Hargreaves, J. M., *An Outline of Otolaryngology as Applied to Aviation Medicine* (Revised), School of Av. Medicine, Randolph Field, Texas, 1939.

Junker, H., " Influence of Heterophoria on Binocular Depth Perception," *Arch. f. Ophth.*, 1940, cxlii, 367–388 (*Abstract Arch. Ophth.*, June, 1942).

Kronfeld, A., " Eine experimentall-psychologische Tauglichkeitsprüfung zum Flugdienst," *Ztschr. f. angew. Psychol.*, Leipzig, 1919, xv, 193–235.

Livingstone, P. C., *Manual*, ccxlvii, No. 6306, p. 37.

Lythgoe, R. J., " Mechanism of Dark Adaptation ; Critical Résumé," *Brit. Journ. Ophth.*, 1940, xxiv, 21–43.

Mandelbaum, J., " Dark Adaptation," *Arch. Ophth., New York*, 1941, xxvi, 203.

Marshall, G. S., " Respiration in High Flying, *Proc. Roy. Soc. Med.*, 1937, xxx, 9–20.

McFarland, R. A., and Barach, A. L., " The Response of Psycho-neurotics to Variations in Oxygen Tension," *Amer. Journ. Psychiat.*, 1937, xciii, 1315-1341.

McFarland, R. A., " The Psychological Effects of Oxygen Deprivation (Anoxaemia) on the Human Behaviour," *Arch. Psychol.*, 1932, No. 145.

McGibbon, J. E. G., *Journ. Laryn. and Otol.*, 1942, lvii, 344–350.

McFarland, R. A., and Evans, J. N., "Alterations in Dark Adaptation under Reduced Oxygen Tensions," *Amer. Journ. Physiol.*, 1939, cxxvii, 37–50.

Newman, H., and Fletcher, E., "The Effects of Alcohol on Vision," *Amer. Journn. Med. Sci.*, 1941, ccii, 723–731.

Poppen, J. R., "Aerial Equilibration," *Journ. Aviation Med.*, 1934, v, 96–103.

Ruff, Siegfried, and Strughol, Hubertus, *Compendium of Aviation Medicine* (translated under the sponsorship of the Committee on Aviation Medicine of the National Research Council).

Rook, A. F., and Dawson, D. J., "Hypotension and Flying," *Lancet*, 1938, ii, 1503.

Schwichtenberg, A. H., "The Evaluation of Orthoptic Training for Aviators," *Amer. Journ. Ophth.*, 1938, xxi, 980–990.

Schubert, G., *Physiologie des Menschen im Flugzeug*, Springer, Berlin, 1935.

Schroeder, R. W. (*see* McFarland, R.A.), "The Psychological Effects of Oxygen Deprivation (Anoxaemia) on Human Behaviour."

Young, C. A., *Hypotension in Aviation War Medicine*, p. 375, Philosophical Library, New York, 1940.

## Figures and Tables

**Figure 1. f1:**
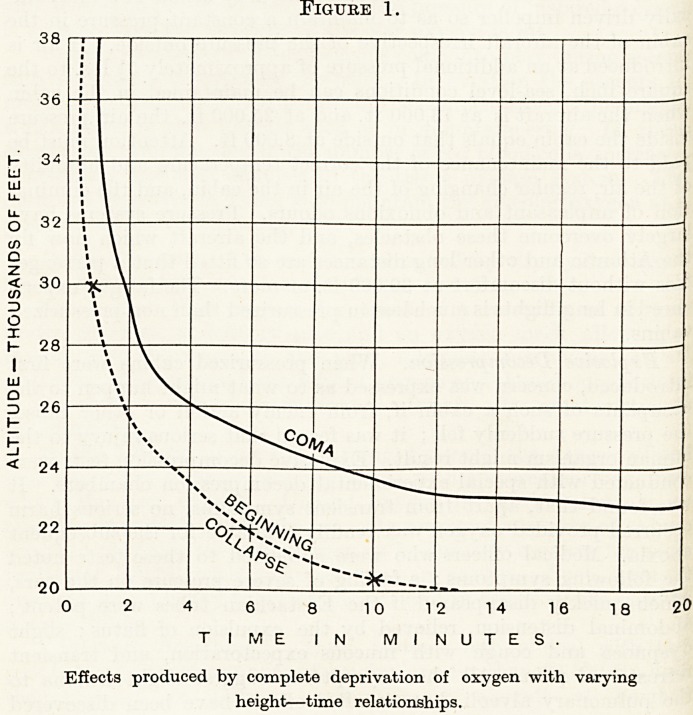


**Figure 2. f2:**